# A low dosage of the dopamine D2-receptor antagonist sulpiride affects effort allocation for reward regardless of trait extraversion

**DOI:** 10.1017/pen.2020.7

**Published:** 2020-06-23

**Authors:** Hanno Andreas Ohmann, Niclas Kuper, Jan Wacker

**Affiliations:** 1 Faculty of Psychology and Human Movement Science, Universität Hamburg, Hamburg, Germany; 2 Faculty of Psychology and Sports Science, Universität Bielefeld, Bielefeld, Germany

**Keywords:** Sulpiride, Dopamine, Approach motivation, EEfRT, Trait extraversion

## Abstract

Dopamine (DA) is known to be involved in various aspects of reward processing and goal-directed behavior. The present preregistered study aims at directly accessing the causal influence of DA activity on reward motivation in humans, while also accounting for trait extraversion. Therefore, we examined the effect of a single dose of the DA D2 receptor antagonist sulpiride (200 mg) on effort allocation in a modified version of the Effort-Expenditure for Reward Task (EEfRT). Based on its presumably DA increasing action, we expected the low dose of sulpiride to increase participants’ willingness to allocate effort during the modified EEfRT relative to placebo, especially in trials with low probability of reward attainment. Further, we expected a moderating effect of trait extraversion on the effects of sulpiride. Two hundred and three healthy male participants were tested in a randomized, double-blind between-subjects design. Contrary to our expectations, sulpiride reduced the average number of clicks within the modified EEfRT and did not interact with reward attributes, suggesting a more global and not reward-specific effect of sulpiride. Furthermore, trait extraversion did not moderate the effect of sulpiride. Our results provide initial support for the validity of the modified version of the EEfRT, suggesting a possible inhibiting effect of a low dose of sulpiride on approach motivation regardless of trait extraversion. However, given the mixed pattern of findings and the possible confounding role of motoric abilities, further studies examining these effects are clearly warranted.

Deciding to go for a possible reward requires evaluating its positive and negative consequences and has been described as a complex “cost-benefit analysis” (Phillips, Walton, & Jhou, 2007). This analysis is based on comparing the positive aspects of the benefits, mainly the reward magnitude (Depue & Collins, 1999) and the aversive aspects of the costs, for example, time spent to achieve the reward or the risk of not achieving it at all (Chong et al., 2015; Hauber & Sommer, 2009). Phillips et al. (2007) suggested a model in which both aspects (costs and benefits) can be described as a hyperbolic function in which a specific threshold has to be reached, so that the behavior to reach the goal is initiated. This threshold is not a fixed point, it varies between situations (e.g., based on the availability of alternatives) and individuals (Walton, Kennerley, Bannerman, Phillips, & Rushworth, 2006; Zald & Treadway, 2017). On a neurophysiological level, dopamine (DA) has been ascribed a central role in this cost-benefit analysis. DA is the predominant catecholamine neurotransmitter inside the brain (Baik, 2013), and the central reward pathway of the brain is the mesocorticolimbic dopamine system (MCLDA). Its widespread neural connections range from the ventral tegmental area and the ventral striatum to regions of the prefrontal cortex (Brooks & Berns, 2013) as well as the anterior cingulate (Hickey, Chelazzi & Theeuwes, 2010). DA binds to a wide range of different receptor types (D1 to D5). Activation of D1 and D5 receptors leads to an excitatory neural reaction and activation of D2, D3 and D4 receptors leads to an inhibitory neural reaction (Serra et al., 1990). Furthermore, the location of the DA receptor (pre-/postsynaptic) alters the effect of its activation (Missale, Nash, Robinson, Jaber, & Caron, 1998; Serra et al., 1990). DA is activated via motivational aspects of approach and avoidance situations (Berridge & Kringelbach, 2008; Salamone, Correa, Mingote, Weber, & Farrar, 2006). According to Depue & Collins (1999), DA promotes goal-directed behaviour and initiation of motor activity, increasing the preference of behavioral options with the highest possible benefit (Nicola, Hopf, & Hjelmstad, 2004; Treadway & Zald, 2011; Walton et al., 2006). According to Phillips et al. (2007), the DA level within the mesolimbic brain structures should directly affect the individual cost-benefit analysis. This assumption is supported by studies in animals (Salamone, Correa, Yang, Rotolo, & Presby, 2018). Pharmacological reduction of DA levels in rodents reduces the willingness to choose behavioral options with high costs and high rewards (Salamone et al., 2016; Yohn et al., 2015), while increasing DA levels has opposite effects (Salamone et al., 2016; Sommer et al., 2014). Moreover, rodents with impaired DA functioning show deficits in their goal-directed behavior (Cannon & Palmiter, 2003; Zhou & Palmiter, 1995).

In humans, DA activity can be modulated using systemic administration of dopaminergic agents like the selective DA D2 receptor antagonist sulpiride. D2 receptors can be found widespread in various structures of the brain such as the prefrontal, cingulate, temporal, and enthorinal cortex as well as the amygdala and the hippocampus, but highest concentrations of D2 receptors can be found in mesolimbic brain structures, such as the striatum and the nucleus accumbens (NAcc; Missale et al., 1998; Beaulieu, Gainetdinov, & Sibley 2011). Therefore, sulpiride is thought to mainly affect functions of the MCLDA, which is in line with studies investigating sulpiride’s clinical effects in the treatment of various mental disorders, for example, depression (Serra et al., 1990; Kuroki, Meltzer, & Ichikawa, 1999), chronic fatigue syndrome (Pardini et al., 2011), or schizophrenia (Miyamoto, Duncan, Marx, & Lieberman, 2005; Lai, Chang, Kao Yang, Lin, & Lin, 2012). However, the intake of sulpiride can have complex effects, which are thought to partly depend on the location of the D2 receptors (pre-/postsynaptic) it primarily blocks (Ford, 2014): low doses of sulpiride are believed to mainly block presynaptic D2/D3 autoreceptors (Kuroki et al., 1999; Mereu, Casu, & Gessa, 1983), resulting in increased DA release and DA synthesis in some parts of the brain (Tagliamonte et al., 1975; Rankin et al., 2009), which might explain its antidepressant effects (Serra et al., 1990; Kuroki et al., 1999). On the other hand, high doses of sulpiride are believed to mainly block postsynaptic D2 receptors thereby lowering DA release and signaling in the brain (Eisenegger et al., 2014; Boschen, Andreatini, & da Cunha, 2015). High doses are therefore used as an antipsychotic drug in patients with schizophrenia (Miyamoto et al., 2005; Lai et al., 2012). Both effects are not completely separable and may occur both in different time frames depending on the precise dose of sulpiride (Mueller, Makeig, Stemmler, Hennig, & Wacker, 2011).

Furthermore, the effects of sulpiride have repeatedly been shown to vary considerably between individuals depending on traits thought to be partly based on individual differences in DA. For example, extraversion is thought to be related to differences in reward processing (Smillie, 2013) and approach motivation in general (Elliot & Thrash, 2002) based on individual differences in DA functioning (Depue & Collins, 1999; Wacker & Smillie, 2015). And indeed, several studies observed that extraversion and related traits completely reversed the effects of sulpiride on various behavioral and neurophysiological measures (Chavanon, Wacker & Stemmler, 2013; Mueller et al., 2014; Wacker, Mueller, Pizzagalli, Hennig, & Stemmler, 2013; Wacker, 2018). For example, sulpiride reduced performance of relatively extraverted and increased performance of relatively introverted participants on a working memory task (Chavanon, Wacker, Leue, & Stemmler, 2007) and a virtual ball-catching task (Mueller et al., 2014). This pattern has been explained by an inverted-U function linking DA activity to behavior with medium DA activity resulting in optimal performance and both extraversion and sulpiride having an impact on DA levels (Chavanon et al., 2013).

Therefore, traits related to DA functioning should always be considered when examining the effects of sulpiride. Studies investigating sulpiride’s direct effect on reward processing in humans using behavioral measures are sparse, often focusing on effects of higher dosages of sulpiride (>400 mg). While, for example, Kahnt, Weber, Haker, Robbins, & Tobler (2015) failed to find behavioral changes after the intake of 400 mg amisulpiride within an outcome prediction task, Diederen et al. (2017) found that participants’ ability to predict future rewards was reduced after intake of 600 mg sulpiride. Ojala et al. (2018) reported that the intake of 400 mg of sulpiride reduced healthy participants overweighting of low probabilities within a decision-making task, resulting in less risky choices. Weber et al. (2016) found that 400 mg of amisulpiride reduced participants’ motivation to choose immediate rewards in a delay-discounting task. Direct evidence for dosage-dependent effects of sulpiride comes from a study conducted by Eisenegger et al (2014). They found that 800 mg sulpiride did not disrupt reward learning within a reinforcement learning task, but reduced number of correct choices of healthy participants only for possible gains not for losses. This effect was directly associated with the serum sulpiride level measured via blood samples, indicating greater impairments are associated with higher serum sulpiride levels. Overall, literature examining sulpiride’s main effects on reward processing and approach motivation hints at inhibiting/disrupting effects of higher dosages of sulpiride. However, most studies had small sample sizes, used different tasks, and focused on different aspects of reward processing, reducing generalizability of results and leaving us with a somewhat unsatisfying and incomplete understanding of sulpiride’s effects on reward motivation in human. Effects of lower dosage sulpiride on reward processing in humans remain even more unclear.

The sparse literature on sulpiride’s effect on reward motivation makes the choice of an appropriate task especially important. One task that has been successfully employed to examine individual differences in reward/approach motivation is the Effort Expenditure for Rewards Task (EEfRT, Treadway, Buckholtz, Schwartzman, Lambert, & Zald, 2009), which is based on a concurrent choice paradigm developed by Salamone, Cousins, & Bucher (1994) to explore effort-based decision-making in rodents. The original EEfRT measures individual differences in human reward processing by having participants decide between high-cost/high-reward (hard task) and low-cost/low-reward (easy task) behavioral options. The tendency to choose the hard task rather than the easy task has been shown to be associated with high levels of approach motivation, as measured,for example, via personality questionnaires (Geaney, Treadway, & Smillie, 2015). According to Smillie (2008), trait behavioral activation system (BAS) sensitivity and extraversion should be predominantly related to reward sensitivity, while trait behavioral inhibition system (BIS) sensitivity and neuroticism should be predominantly related to punishment sensitivity. The EEfRT introduces a reward context, and one would, thus, expect relatively specific associations between behavior in the task and extraversion/trait BAS sensitivity (and their low pole, which is associated with anhedonia; Rizvi, Pizzagalli, Sproule & Kennedy, 2016; Mueller, Panitz, Pizzagalli, Hermann & Wacker, 2015). The mere absence of a reward should have a much smaller impact compared to tasks that introduce a strong punishment (e.g., deduction of money). Healthy participants’ preference for the hard task has further been shown to correlate with lower scores on negative affect, depressive symptoms, and anhedonia (Treadway et al., 2009). Recently, we were able to demonstrate that left frontal anodal transcranial direct current stimulation over the dorsolateral prefrontal cortex increased participants’ willingness to choose the hard task depending on reward attributes (Ohmann, Kuper & Wacker, 2018), which is in line with models associating left frontal brain activity with approach motivation (Harmon-Jones & Gable, 2017; Rutherford and Lindell, 2011). Wardle, Treadway, Mayo, Zald, & de Wit (2011) were the first to show that the EEfRT is also sensitive to pharmacological manipulation of DA, as d-amphetamine increased participants’ overall effort allocation. Further evidence comes from patients suffering from impaired approach motivation: patients with schizophrenia (Fervaha, Foussias, Agid, & Remington, 2013; Barch, Treadway, & Schoen, 2014; McCarthy, Treadway, Bennett, & Blanchard, 2016), first-episode psychosis (Chang et al., 2019), and depression (Treadway, Bossaller, Shelton, & Zald, 2012; Yang et al., 2014) were less willing to choose the hard task as compared to healthy controls, indicating patients’ impaired approach motivation. Furthermore, the number of hard task choices was found to be negatively correlated with the severity of anhedonic symptoms in patients with schizophrenia (e.g., Barch et al., 2014) as well as in patients with depression (e.g., Yang et al., 2014). Anhedonia is closely linked to dysregulated DA functioning within the MCLDA (Pizzagalli et al., 2009; Heshmati & Russo, 2015), which is thought to play a central role in participants’ effort allocation. Supporting this notion, Huang et al. (2016) found that the number of hard task choices was directly linked to the activity of the NAcc, which is a key structure of the MCLDA, in both patients with schizophrenia and healthy participants.

Taken together, the EEfRT is a well-established indicator of individual differences in approach motivation thought to be based in individual differences in MCLDA functioning. Thus, manipulating the activity of the MCLDA (e.g., via administration of sulpiride) should influence participants’ performance in the EEfRT.

## Hypotheses

The main hypotheses and analyses were preregistered at the Open Science Framework on August 9, 2017 after the collection of 70 datasets and before accessing any of the data included in the current analyses (https://osf.io/e5fn9).

### Substance group and extraversion

First, we expected a main effect of substance group (sulpiride 200 mg vs. placebo) on approach motivation, which is assessed in terms of individual’s willingness to allocate more effort in return for greater rewards within the EEfRT. Second, conceptually replicating prior work on extraversion and DA using pharmacological manipulations of DA, we expected an interaction of extraversion and substance group in the prediction of approach motivation. Previous research has shown that the individual level of trait extraversion is linked to the neuronal and behavioral effects of sulpiride (e.g., Chavanon et al., 2013; Mueller et al., 2014; Wacker et al., 2013; Wacker, 2018), with opposite effects in individuals high versus low in extraversion, possibly due to an inverted-U function linking DA functioning and behavior and certain neural measures. Expecting to observe similar effects using the EEfRT as dependent variable, we predicted that extraversion should increase effort expenditure for individuals low in extraversion and reduce effort allocation in individuals high in extraversion within the sulpiride group as compared to the placebo group.

### EEfRT – task validity

For our modified version of the original EEfRT (Treadway et al., 2009; see 2.5 for details on all modifications), we expected effects comparable to the original. We expected the reward attributes (reward magnitude and probability of reward attainment) to be positive predictors of the average number of clicks, whereas we expected trial number (e.g., an indicator of fatigue) to be a negative predictor of the average number of clicks within our modified version of the EEfRT. We only tested right-handed participants, who typically show worse motoric performance with their left hand on finger tapping tests (Hervé, Mazoyer, Crivello, Perchey, & Tzourio-Mazoyer, 2005). Therefore, we expected the factor hand to be a significant predictor of the average number of clicks, as participants should make fewer clicks with their left hand due to the increased effort requirements. As our modifications might have increased the influence of motoric abilities and this is the first study to use these modifications, we ran a pre-analysis to see whether higher motoric abilities are associated with greater average number of clicks throughout the actual task and should therefore be statistically controlled for in the analyses.

### Secondary analysis

To further validate our modified EEfRT, we analyzed the correlations between extraversion, BIS/BAS, and effort allocation in the task. Previous research has shown positive correlations between BAS and task performance on the original EEfRT in trials with low probability of reward attainment (Geaney et al., 2015), which we also expected for our modified version. To probe the specificity of these effects, we exploratorily correlated effort expenditure with other personality variables. Finally, we checked for the effect of previous trial feedback based on research by Anand, Oehlberg, Treadway, & Nusslock (2016), who found that feedback of a previous task impacted participants’ performance in the following EEfRT task. Comparable to Anand et al. (2016), we expected negative feedback within the previous trial (no money won) to reduce participants’ average number of clicks as compared to positive feedback (money won). Additionally, we checked for possible moderating effects of substance group and extraversion on feedback in an exploratory fashion. As previous trial feedback is not present in the first trial of each block, these two trials were excluded from these analyses.

## 1. Methods

### 1.1 Participants

We recruited right-handed, physically and psychologically healthy male participants aged 18–35 years using online notice boards, advertising via blackboard postings, flyers, and recruiting booths at local universities (University of Hamburg, Germany; Helmut-Schmidt University, Germany; Hochschule für Angewandte Wissenschaften Hamburg, Germany). Out of 210 recruited participants, 7 had to be excluded for different reasons (4 showed uncompliant behavior, e.g., not following instructions; 2 were unable to swallow the capsule; 1 dataset was lost due to technical failure). Thus, the final sample consisted of 203 participants (age: *M* = 25.10; *SD* = 3.94), 102 participants within the sulpiride group and 101 participants within the placebo group. As can be seen in Table 1, participants of the two substance groups did not differ in any of the main demographic variables assessed before the testing session (age, BMI, fluid and crystallized IQ, BIS/BAS; extraversion). The significant difference in motoric trials assessed while participants were under the influence of placebo/sulpiride is discussed in Section 1.5.

**Table 1.** Simple *t*-test comparisons of main demographics and covariates for both substance groups

Variable	Sulpiride condition		Placebo condition		*t*	*p*
	*M*	*SD*	*M*	*SD*		
Age	25.32	3.91	24.79	3.99	0.95	0.342
BMI	24.13	2.86	23.93	2.89	0.49	0.624
Fluid IQ	116.56	13.41	113.48	15.69	1.51	0.134
Crystallized IQ	101.29	12.36	101.77	13.55	−0.26	0.793
BIS	17.44	3.35	16.80	3.04	1.42	0.156
BAS	39.82	4.02	39.67	3.81	0.27	0.785
Extraversion	162.28	18.25	161.56	16.89	0.29	0.771
MaxMot-L	118.85	18.39	119.24	21.13	−0.14	0.890
MaxMot-R	131.18	19.15	137.01	19.69	−2.14	**0.034**

BMI = body mass index; fluid IQ = intelligence quotient of fluid intelligence; crystallized IQ = intelligence quotient of crystallized intelligence; BIS = behavioral inhibition system; BAS = behavioral activation system; MaxMot-L= maximum number of clicks within motoric trials exhibited with the left hand; MaxMot-R = maximum number of clicks within motoric trials exhibited with the right hand. Significant effects in bold.

A sensitivity power analysis was carried out to determine our statistical power to detect differences in correlations between extraversion and effort expenditure in the two substance groups. Our statistical power was 80% for the detection of Fisher’s *z*-transformed correlation differences of .40 at *α* = .05. This corresponds, for instance, to a correlation of *r* = .20 in the placebo, and *r* = −.20 in the sulpiride group. Participants received monetary compensation (10€ per hour) and were told that they could gain additional money based on their collected rewards from two computer tasks. At the end of the study, we revealed to all participants that the additional money was fixed at 10€ (which was generally higher than the rewards collected while playing) to ensure equity. The authors assert that all procedures contributing to this work comply with the ethical standards of the relevant national and institutional committees on human experimentation and with the Helsinki Declaration of 1975, as revised in 2008. The study has been approved by the Human Research Ethics Committee of the DGPs (Deutsche Gesellschaft für Psychologie).

We applied strict exclusion criteria to ensure maximum safety for all participants. Exclusion criteria comprised the intake of any kind of prescribed medication over the last 3 months, the consumption of illegal drugs over the last 3 months, the consumption of more than 10 cigarettes per week, high blood pressure (above 140/90) and/or an irregular heartbeat as tested on site using an automatic blood pressure monitor, lifetime medical conditions (in particular epilepsy, endocrinopathies, hypertension, coronary heart disease, bleeding or disease of the bowel, disease of the liver or kidneys), the presence of any mental disorders as diagnosed via DSM-V criteria over the last 3 months (in particular affective, somatoform, psychotic, anxiety, eating, and adaptive disorders, as well as mental disorders triggered by drug abuse) using a standardize clinical interview (Mini-DIPS, Margraf, 1994), and any known allergic reactions to sulpiride or other psychoactive substances.

### 1.2 Manipulation

In a randomized, double-blinded between-subjects design, participants either received a capsule with 200 mg of the DA D2 receptor antagonist sulpiride (e.g., the same dose used by Wacker et al., 2013; Wacker, Chavanon, Stemmler, 2006) or a non-distinguishable placebo. Sulpiride is a substituted benzamide, which is generally well tolerated (Ruther et al., 1999). We decided to use a rather low dosage of 200 mg of sulpiride, because in lower doses sulpiride is believed to have a high affinity to presynaptic autoreceptors and therefore presumably elevates DA levels (Tagliamonte et al., 1975; Rankin et al., 2009) resulting in a mild stimulant effect. This stimulant effect is for example used in the treatment of depression (Serra et al., 1990; Kuroki et al., 1999). This contrasts with higher dosages of sulpiride (>400 mg), which are believed to additionally affect postsynaptic autoreceptors and therefore reducing DA levels, resulting in an overall inhibiting effect (Eisenegger et al., 2014; Boschen et al., 2015) used as an antipsychotic drug in patients with schizophrenia (Miyamoto et al., 2005; Lai et al., 2012). Sulpiride is slowly absorbed from the gastrointestinal tract, with peak serum levels occurring 1 to 6 h after oral ingestion; the average elimination half-life is in the range of 3 to 10 h (Mauri, Bravin, Bitetto, Rudelli, & Invernizzi, 1996).

### 1.3 Randomization

Participants were tested in groups of four (or three, in case one scheduled participant did not appear). Substance condition was assigned with a restricted randomization in order to ensure (1) equal numbers of participants for both conditions and (2) balanced conditions within each group session. For each group testing of four participants, two participants were randomly assigned to the placebo and two to the sulpiride condition based on their assigned identification number. For groups with three participants, the condition originally assigned to the fourth participant’s identification number was dropped.

### 1.4 Procedure

Potential participants took part in a 5-min screening interview (per telephone or in person) which served as a first screening instrument for in- and exclusion criteria. Individuals who appeared eligible for participation were invited into our lab twice (presession and main experimental session).

In the presession, participants received detailed information about sulpiride and the double-blind experimental procedure and gave their informed consent. They were further informed that they were required to refrain from eating and consuming caffeine and nicotine starting 11.5 h (from 22:00 on the day before the test session) before their schedule session. Afterward, the in- and exclusion criteria were checked thoroughly. If all in- and exclusion criteria were met, participants filled out a series of questionnaires, including demographic information, the German BIS/BAS (Strobel, Beauducel, Debener, & Brocke, 2001), and the German NEO-PI-3 (unpublished translation from the NEO-PI-3; Costa & McCrae, 2010) to assess participants’ personality and made an appointment for the main experimental session.

Three or four participants took part in the main experimental session, which started at 9:30. The experimenters inquired whether the participants had indeed refrained from eating and consuming caffeine/nicotine as required and whether they were in good physical condition. Participants then had a standardized, light breakfast and took the assigned capsule. On average, participants started at 9:54 AM (*SD* = 6 min) by completing six computer-based subtests of the Intelligence Structure Battery (INSBAT, Arendasy et al., 2012) to access participants’ fluid and crystallized intelligence. Afterward, participants completed a series of tasks, which are not relevant to the current research question (for further information, see https://osf.io/phr4g). Participants completed the modified EEfRT (see Section 1.5) at 12:55 PM (*SD* = 17 min). After two further tasks, data from which will be reported elsewhere (e.g., Käckenmester, Bott, Wacker, 2019), participants completed a short questionnaire about the surrounding conditions of the lab during the main session, their subjective perceptions of the effects of the sulpiride/placebo intake, and their presumptions concerning the purpose of the study. Finally, participants received written information about the study and their experimental condition (sulpiride or placebo) along with instructions on how to act in case they noticed any side effects, including contact information for emergencies (only sulpiride condition), and were reimbursed for their participation.

### 1.5 Effort-expenditure for reward task

**Figure 1.**
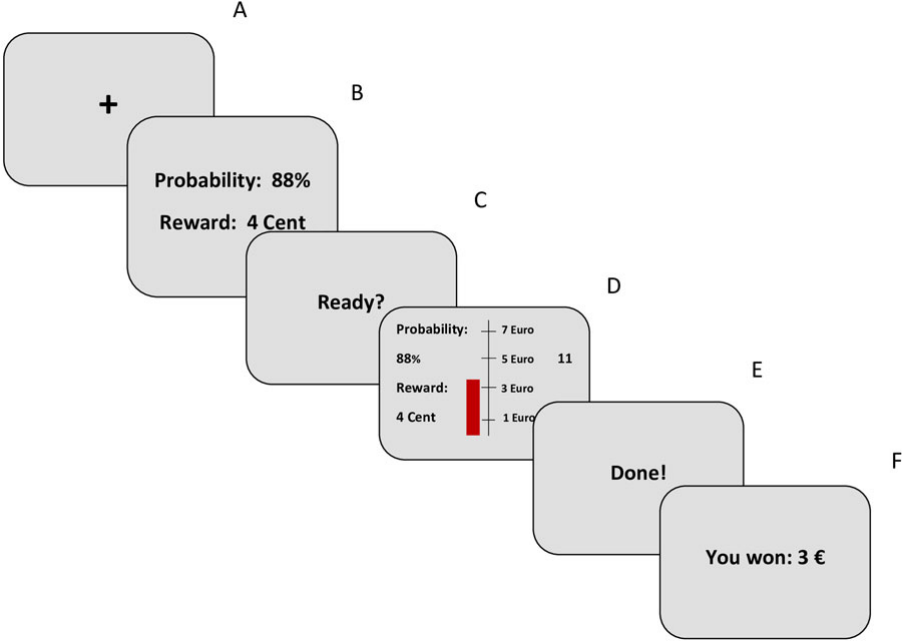
Schematic illustration of one trial of the modified EEfRT. A fixation cross (1s, A) is followed by a screen showing probability of reward attainment and reward magnitude per click for 3s (B). Then, after a ready – screen (1s, C), the main screen for the trial showing a red bar that grows with each click is presented alongside a scale, indicating the current monetary gain and a countdown (20s, D). Finally, task completion is signaled (1,5s, E) and a feedback screen shows the amount of money won (2s, F).

We used a modified and translated (German) version of the EEfRT (Treadway et al., 2009), which was programmed using Presentation software 17.1 (Neurobehavioral Systems Inc, San Francisco). The original EEfRT intends to measure participants’ approach motivation by testing their willingness to exert effort to gain possible monetary rewards. Participants choose between an easy, low-reward task and a hard, high-reward task in every trial. The easy task requires participants to press the space button 30 times in 7 s with their index finger. The hard task requires participants to press the space button 100 times in 21 s with their pinkie finger. The reward for the easy task is fixed to 1€, while the reward for the hard task is variable (ranging between 1.21€ and 4.30€). Furthermore, before the start of each trial, participants are informed about the probability of attaining the reward after successfully completing the trial. The probability is equal for both the easy and the hard task within each trial. In a previous study (Ohmann et al., 2018), we found that using the original EEfRT also comes with a major downside: at least some participants understand that choosing the hard task is often lowering the possible overall monetary gain as the hard task takes almost three times as long as the easy task. Hence, at least some participants’ choices are partly based on a strategic decision and less on approach motivation per se. To overcome this downside, we modified the original EEfRT. First, we fixed the number of trials (2 blocks × 15 trials = 30 trials) and the duration of each trial (= 20 s). Participants use their right hand for one block and their left hand for the other block, and the order of both blocks is randomized. Second, we changed the original choice paradigm. Participants no longer choose between an easy and a hard task. As in the original task, the value of each reward varies, and participants are informed about this at the start of each trial. But instead of presenting specific reward amounts, participants are now presented with a reward amount per click (1/2/3/4/5 cents per click). Thus, participants are able to increase the total possible monetary gain in each trial with each click. In accordance with the original task design, the probability of reward attainment also varied [either 12% (low), 50% (middle), or 88% (high)], which is presented at the start of each trial alongside the reward value per click. Participants were instructed to win as much virtual money as possible throughout the task; however, they were free to choose the amount of effort they exerted in each trial. Critically, the only way to increase the possible monetary gain is to increase the number of clicks in each trial. The task itself is designed close to the original EEfRT but comes with some modifications to prevent the use of strategies (see Figure 1): while pressing the spacebar, a visually presented red bar gradually grows. We implemented a scale (€), so that the participants can always see how much their button presses increase their possible monetary gain. Furthermore, we decided to present the information on the reward amount per click and the probability of attaining the total reward amount in the specific trial throughout the whole trial alongside a countdown (20 s) to increase participants’ awareness of these parameters. After each trial, participants are informed about the amount of money they won during the trial. The order of trials, as well as the probability of reward attainment and the reward magnitudes per click are not randomized between participants, but pre-assigned for each trial. This is done to rule out random feedback differences between participants. Because it is likely that participants with greater motoric ability exert more clicks throughout the task, which does not reflect their actual approach motivation, we included 10 motoric trials (5 at the start of each block, using either the left or the right hand according to the randomized block order) to test participants’ motoric abilities. Within these motoric trials, participants were instructed to press the spacebar as often as possible within 20 s. Critically, participants were not able to gain any rewards in these trials, and visual feedback was reduced to a countdown and a display of the number of clicks they exerted. Participants’ individual motoric abilities were included in our statistical models. Although the inclusion of this factor was not preregistered, we decided to do so, because our preliminary analyses (see Section 2.1) revealed a large impact of participants’ individual motoric abilities on the number of clicks they exerted and participants of both substance groups also differed in their motoric abilities as measured via the motoric trials, as participants within the sulpiride condition did show lower motoric abilities (see Table 1). Therefore, not including this factor could have distorted the results.

### 1.6 Data analysis

Aggregated data were further analyzed using the SPSS 22.0 software (Chicago, IL, USA). Effects on the number of clicks while playing the modified EEfRT were analyzed using Generalized Estimating Equations (GEEs). GEEs are marginal models that allow for robust parameter estimation despite correlated residuals, for example, due to the clustering of trials within participants (Liang, Beaty, & Cohen, 1986; Zeger & Liang, 1986). The models were fit using an exchangeable working correlation matrix. Crucially, they are consistent even when the correlation matrix for the residuals is specified incorrectly. All GEE models included the factors block, trial number, hand, probability (categorical), reward magnitude, the interaction of probability × reward magnitude (sometimes referred to as “expected value”), and participants’ individual motoric abilities. Separate models were computed to test basic predictors of “number of clicks”, the effects of substance group (sulpiride/placebo) on “number of clicks”, as well as interactions between substance group and reward attributes (reward magnitude and probability of reward attainment). Pearson’s correlations were computed between trait variables (BAS/extraversion/etc.) and mean number of clicks within the modified EEfRT adjusted for individual motoric abilities separately for each probability of reward attainment (low/medium/high).

As mentioned above, the main hypotheses and analyses were preregistered at the Open Science Framework on August 9, 2017 after the collection of 70 datasets and before accessing any of the data included in the current analyses (https://osf.io/e5fn9). The results of the other tasks addressed in the preregistration were part of the larger project investigating the effects of DA on behavioral measures and will be reported elsewhere.

## 2. Results

### 2.1 Preliminary analysis – influence of motoric abilities

We ran a pre-analysis to probe whether higher motoric ability is associated with greater average number of clicks throughout the actual task. Therefore, we correlated the data of participants’ motoric trials, both maximum number of clicks (left hand = MaxMot-L; right hand = MaxMot-R) and average number of clicks (left hand = AvMot-L; right hand = AvMot-R) with the average number of clicks within the actual task. Participants on average exerted a maximum of 134.08 (*SD* = 19.59) clicks within motoric trials with their right hand and a maximum of 119.04 (*SD* = 19.75) clicks on motoric trials with their left hand, and the difference between both hands was significant (*t*(202) = 11.41, *p* < .001). MaxMot-L showed significant positive correlations with the average number of clicks within trials exerted with the left hand (*r* = .585, *p* < .001) as well as within trials exerted with the right hand (*r* = .580, *p* < .001). MaxMot-R also showed significant positive correlations with the average number of clicks within trials with the left hand (*r* = .477, *p* < .001) as well as with trials with the right hand (*r* = .648, *p* < .001). The pattern for the average number of clicks within motoric trials was highly comparable and including the average number of clicks within motoric trials in the main analyses instead did not change the results in a significant way.

### 2.2 Preliminary analysis – reliability of the EEfRT

We calculated the reliability of the mean number of clicks. Therefore, the mean number of clicks of each participant was residualized on his motoric abilities. Reliability estimates were obtained by correlating the adjusted number of clicks of both hands (out of two blocks, one was conducted with the right hand and one with the left hand) and applying a Spearman’s brown correction to the resulting estimate. The overall mean number of clicks showed a high correlation between both hands (Rel = .78). When separately analyzing the data for each probability and reward amount per click, correlations ranged between Rel = .67 and Rel = .87. The overall high correlations indicate a robust reliability of the modified EEfRT.

### 2.3 Validity of the modified EEfRT

**Figure 2.**
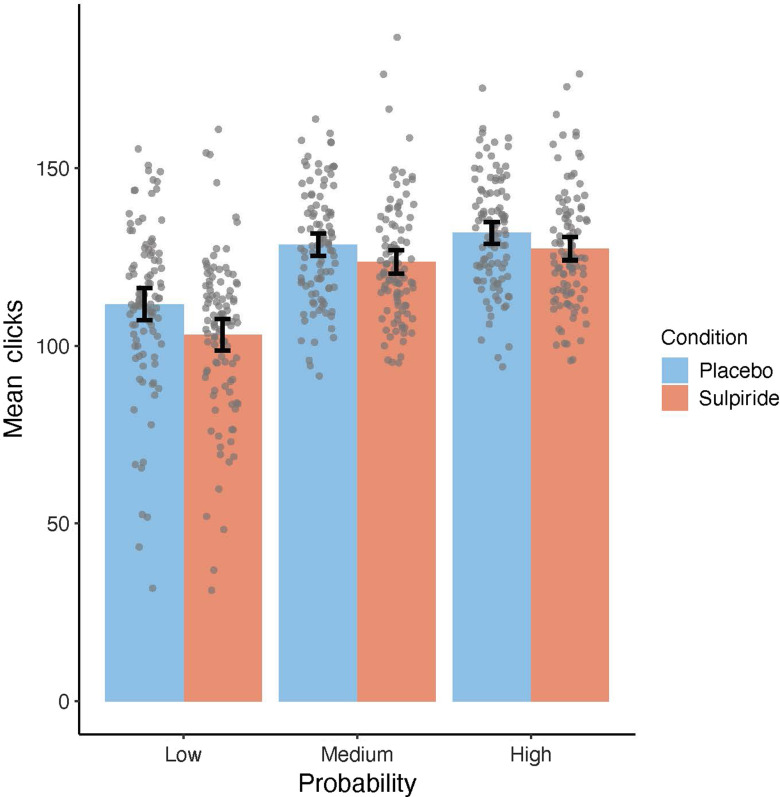
Mean number of clicks adjusted for participants’ motoric abilities, comparing trials with low probability of reward attainment (left), medium probability of reward attainment (middle), and high probability of reward attainment (right) as compared between both substance groups (sulpiride groups is shown in red and placebo group is shown in blue). Data points are added as dots for individual scores. Error bars depict a 95% confidence interval (CI) of the mean.

**Figure 3.**
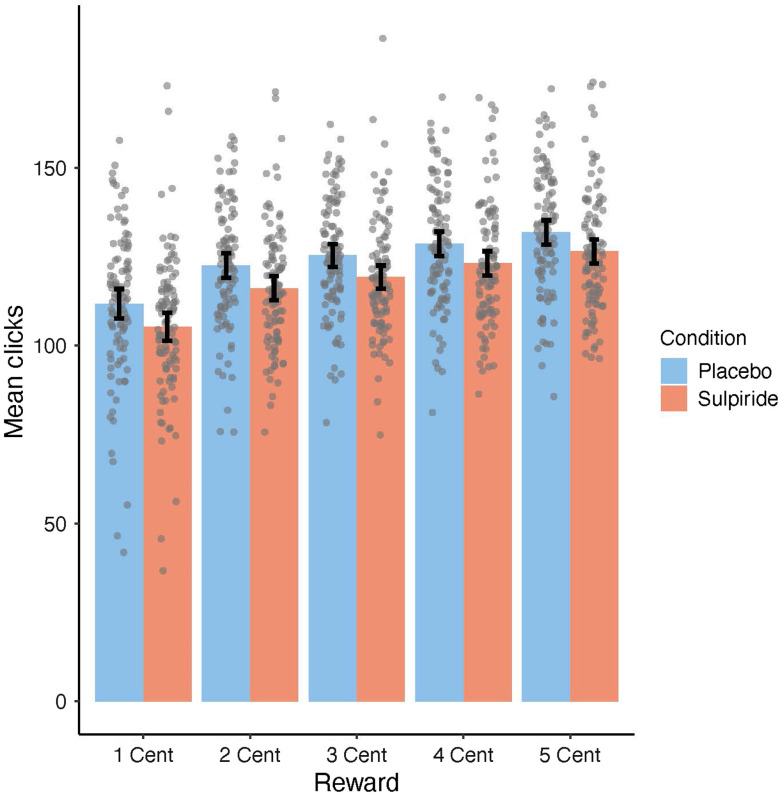
Mean number of clicks adjusted for participants’ motoric abilities, comparing trials with different reward magnitude per click, ranging from 1 cent (most left) to 5 cent (most right) as compared between both substance groups (sulpiride groups is shown in red and placebo group is shown in blue). Data points are added as dots for individual scores. Error bars depict a 95% confidence interval (CI) of the mean.

Participants on average exerted 121.00 clicks per trial (*SD* = 16.70; range = 76.43–172.37). We conducted a series of GEE models to test the validity of the modified EEfRT (see Table 2, GEE model 1–4). GEE model 1 examined main effects of task-dependent variables (reward magnitude, probability of reward attainment, and trial number) as well as four variables unique to our study (block, hand, MaxMot-L, and MaxMot-R) on the average number of clicks. In line with previous studies, significant positive main effects were found for probability (see Figure 2) and reward magnitude (see Figure 3), and a significant negative main effect was found for trial number, indicating that all three factors were predictors of average number of clicks (all *p*s < .001). The factor block did not reach significance (*β* = −1.52, *χ* ^2^(1) = 3.22, *p* = .073), indicating that there were no appreciable differences in average number of clicks between both blocks. In accordance with our previous work with the original EEfRT, the factor hand reached significance (*β* = 11.99, *χ*²(1) = 0.90, *p* < .001), indicating that participants clicked the button more often with their right hand.

**Table 2.** GEE models for basic predictors of average number of clicks (EEfRT)

Effect	*β*	*se*	*χ*²	*p*
Model 1				
Reward magnitude	5.11	0.40	160.13	**<.001**
Probability 50%^a^	17.10	1.69	102.26	**<.001**
Probability 88%^a^	25.36	1.86	185.97	**<.001**
Probability 50%^a^ × Reward magnitude	0.37	0.36	1.07	.302
Probability 88%^a^ × Reward magnitude	−1.04	0.35	8.58	**.003**
Trial	−0.26	0.04	45.73	**<.001**
Hand	11.99	0.85	199.49	**<.001**
Block	−1.52	0.85	3.22	.073
MaxMot-L	0.43	0.15	8.12	**.004**
MaxMot-R	0.22	0.11	4.19	**.041**
Model 2				
MaxMot-L × Hand	−0.16	0.08	3.52	.061
MaxMot-R × Hand	0.18	0.06	8.35	**.004**
Model 3				
MaxMot-L × Reward	−0.01	0.02	2.38	.683
MaxMot-R × Reward	0.03	0.02	0.17	.123
Model 4				
MaxMot-L × Probability 50%^a^	−0.01	0.06	0.05	.817
MaxMot-L × Probability 88%^a^	−0.06	0.07	0.75	.386
MaxMot-R × Probability 50%^a^	0.07	0.08	0.84	.359
MaxMot-R × Probability 88%^a^	0.09	0.08	1.26	.262

*Note*. All models included probability (categorical), reward magnitude, trial number, block, hand as within-subjects variables, and MaxMot-L and MaxMot-R as between subject variables; *χ*² = Wald chi-square; *β* = regression coefficient; significant effects in bold.

a Reference category: 12% probability.

MaxMot-L and MaxMot-R both reached significance, indicating that participants with greater motoric ability as measured within the motoric trials also exerted more clicks within the modified EEfRT, which is in line with the pre-analysis we conducted showing significant positive correlations (see Section 2.1). Model 2 revealed a significant interaction of hand used within the task and MaxMot-R (*β* = 0.18, *χ*²(1) = 8.35, *p* = .004), whereas the analogous interaction failed to reach significance for MaxMot-L (*β* = −0.16, *χ*²(1) = 3.52, *p* = .061). As both models indicate motoric abilities to influence task performance (supporting our preliminary analysis, see Section 2.1), we decided to include both factors as well as their interactions with the hand used within the task in all following models. Two additional GEE models (see Table 2, model 3–4) tested possible interactions of participants’ motoric ability and reward attributes. None of these interactions reached significance, indicating that participants’ motoric abilities did not affect task performance depending on reward attributes.

### 2.4 Effects of sulpiride and extraversion

**Figure 4.**
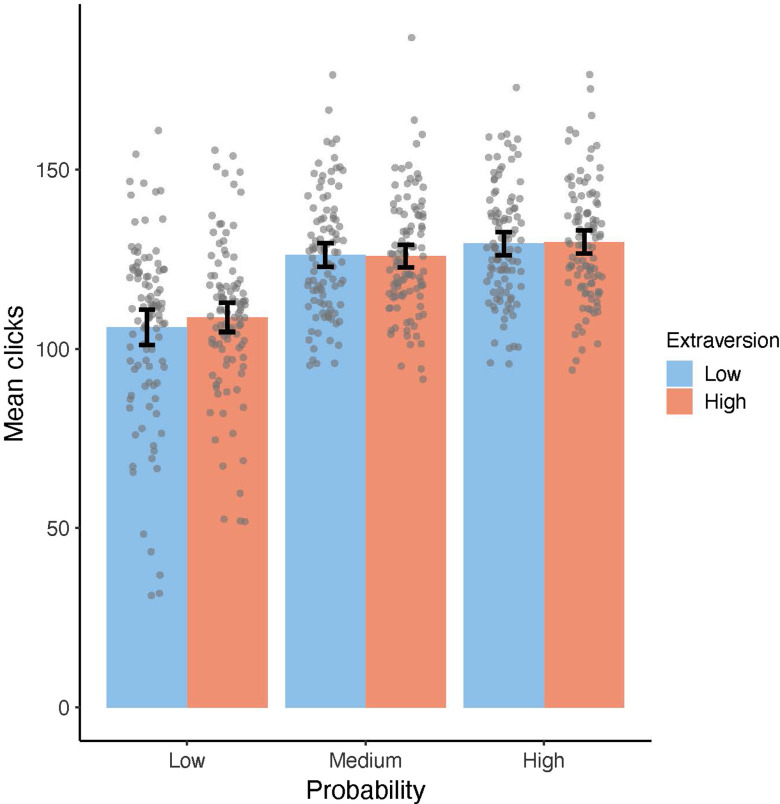
Mean number of clicks adjusted for participants’ motoric abilities, comparing trials with low probability of reward attainment (left), medium probability of reward attainment (middle), and high probability of reward attainment (right) as compared between participants with high extraversion (shown in red) and participants with low extraversion (shown in blue). Extraversion categories were obtained by splitting data into two equally sized groups. Data points are added as dots for individual scores. Error bars depict a 95% confidence interval (CI) of the mean.

Overall, three GEE models were computed to test the effects of sulpiride on the average number of clicks within the modified EEfRT (see Table 3, GEE model 5–7). In model 5, we tested the main effect of substance group on the average number of clicks, which reached significance (*β* = −4.16, *χ*²(1) = 7.26, *p* = .007), indicating that participants showed a reduced average number of clicks under sulpiride. In model 6 and 7, we analyzed the interaction of substance group with reward magnitude and probability and found that the effect of sulpiride on average number of clicks was not moderated by either of the two factors (see Figure 2 and 3). Three further GEE models (see Table 3, GEE model 8–10) tested the effect of trait extraversion on average number of clicks within the modified EEfRT. Model 8 revealed a main effect of extraversion (*β* = 1.71, *χ*²(1) = 4.52, *p* = .034), indicating that participants with higher trait extraversion exerted a higher average number of clicks. While model 9 revealed no significant interaction of extraversion and reward magnitude, model 10 revealed a significant interaction of extraversion and probability. Comparing low versus high probability (*β* = −3.13, *χ*²(1) =, *p* = .027) as well as comparing low and medium probability trials (*β* = −3.21, *χ*²(1) =, *p* = .041) revealed significant interactions with extraversion, due to participants with higher extraversion being less affected by the probability of reward attainment than participants with lower extraversion (see Figure 4). In GEE model 11, we tested for a possible interaction of substance group and trait extraversion, which was not significant. GEE model 12 was computed to test for a possible interaction of substance group and participants’ motoric abilities, which was also not significant, indicating that sulpiride did not alter the association between average number of clicks within the modified EEfRT and motoric abilities.

**Table 3.** GEE models of substance group and extraversion effects on average number of clicks (EEfRT)

Effect	*β*	*se*	*χ²*	*p*
Model 5				
Substance	−4.16	1.54	7.26	**.007**
Model 6				
Substance	−5.12	2.49	4.21	**.040**
Substance × Reward Magnitude	0.32	5.67	0.32	.573
Model 7				
Substance	−6.76	2.69	6.34	**.012**
Substance × Probability 50%^a^	3.69	2.33	2.51	.113
Substance × Probability 88%^a^	4.12	2.63	2.46	.117
Model 8				
Extraversion	1.71	0.80	4.52	.**034**
Model 9				
Extraversion	1.10	1.14	0.94	.333
Extraversion × Reward Magnitude	0.20	0.28	0.53	.468
Model 10				
Extraversion	3.82	1.53	6.22	**.013**
Extraversion × Probability 50%^a^	−3.21	1.45	4.20	**.027**
Extraversion × Probability 88%^a^	−3.13	1.53	4.90	**.041**
Model 11				
Substance	−4.27	1.52	7.92	**.005**
Extraversion	1.33	1.16	1.32	.252
Substance × Extraversion	0.83	1.54	0.29	.593
Model 12				
Substance	−20.28	14.89	1.86	.173
Substance × MaxMot-L	0.26	0.18	2.12	.145
Substance × MaxMot-R	−0.11	0.15	0.55	.457

*Note*. All models included probability (categorical), reward magnitude, trial number, block, hand as within-subjects variables, and MaxMot-L and MaxMot-R as between subject variables; *χ*² = Wald chi-square; *β* = regression coefficient; significant effects in bold.

a Reference category: 12% probability.

### 2.5 Secondary analyses

To further validate our modified EEfRT, we correlated the average number of clicks within the task (adjusted for participants’ individual motoric abilities, see section 2.1) with the personality traits extraversion and BIS/BAS. Several other personality traits were exploratorily included to test the specificity of the effects (see Table 4). As expected, trait BAS correlated positively with the adjusted number of clicks within trials with low probability of reward attainment mirroring the pattern observed for extraversion. The correlation between extraversion and the adjusted number of clicks did not differ significantly between the substance groups (placebo: *r* = .09, sulpiride: *r* = .21, *Z*_diff_ = 0.86, *p* = .391). These findings were similar for trait BAS (placebo: *r* = .09, sulpiride: *r* = .32, *Z*_diff_ = 1.71, *p* = .086). However, openness also correlated positively with the adjusted number of clicks within trials with low probability of reward attainment. None of the other personality traits correlated significantly.

**Table 4.** Zero-order correlations between EEfRT average number of clicks and trait variables

Trait variable	Reward probability		
	12%	50%	88%
BAS	0.212**	0.124	0.103
BIS	−0.081	0.081	0.052
Extraversion	0.193**	0.051	0.054
Openness	0.159*	0.039	0.024
Conscientiousness	0.113	0.033	0.023
Neuroticism	−0.093	0.072	0.062
Agreeableness	−0.052	0.035	−0.016

*Note.* EEfRT = Effort Expenditure for Rewards Task; BAS = behavioral activation system; BIS = behavioral inhibition system scale; significant effects in bold. * *p* < .05 ** *p* < .01.

Additionally, based on Anand et al.’s (2016) findings, who used the original EEfRT, we tested for a possible impact of feedback. But instead of investigating previous task feedback, we tested for the impact of previous trial feedback (money won vs. no money won in the previous trial). We computed three additional GEE models (13–15, see Table 5). GEE model 13 revealed a highly significant impact of previous trial feedback, indicating that negative feedback significantly reduced the average number of clicks within the task as compared to positive feedback. GEE model 14 and 15 were conducted in an exploratory fashion, to check for a possible moderating effect of substance group and extraversion. Both models revealed that the effect of previous trial feedback on the average number of clicks was neither moderated by substance group nor by extraversion.

**Table 5.** GEE models of feedback effects on average number of clicks (EEfRT)

Effect	*β*	*se*	*χ²*	*p*
Model 13				
Feedback	2.27	0.35	41.54	**<.001**
Model 14				
Feedback	2.15	0.53	16.25	**<.001**
Substance	−6.97	2.87	5.90	**.015**
Substance × Probability 50%	3.94	2.40	2.69	.101
Substance × Probability 88%	4.17	2.69	2.40	.121
Substance × Feedback	0.24	0.80	0.09	.762
Model 15				
Feedback	2.27	0.35	42.49	**<.001**
Extraversion	3.43	1.59	4.67	**.031**
Extraversion × Probability 50%	−3.14	1.47	4.56	**.033**
Extraversion × Probability 88%	−3.01	1.55	3.79	.051
Extraversion × Feedback	0.57	0.36	2.50	.114

*Note*. All models included probability (categorical), reward magnitude, trial number, block, hand as within-subjects variables, and MaxMot-L and MaxMot-R as between subject variables; *χ*² = Wald chi-square; *β* = regression coefficient; significant effects in bold.

a Reference category: 12% probability.

## 3. Discussion

In the present study, we aimed to (1) validate our new modified version of the EEfRT (Treadway et al., 2009) as a measure of DA-based approach motivation and (2) conceptually replicate the modulating effect of extraversion on a pharmacological manipulation of DA using the modified EEfRT as a dependent variable more directly tapping into approach motivation than the variables examined in previous studies (e.g., Wacker, 2018; Wacker et al., 2013). We will discuss the implication of the current findings for each of these goals.

### 3.1 Validity of the modified version of the EEfRT

Supporting the validity of our modified task, the pattern of effects of reward, probability, trial, hand, and block on the average number of clicks closely matched the pattern typically observed for the original EEfRT. Previous studies investigating the psychometric properties of the original EEfRT in a clinical sample (schizophrenic patients) and a healthy control sample found robust reliability, exceeding the reliability of four other effort-based decision-making tasks (Reddy et al., 2015; Horan et al., 2015). Our results indicate that the reliability of the modified EEfRT is also high. Furthermore, we observed positive correlations between extraversion, trait BAS as well as openness and the number of clicks only in trials with low probability of reward attainment. This is in line with studies indicating that especially trials with low probability of reward attainment are associated with different indicators of approach motivation (e.g., Geaney et al., 2015). The fact that some studies failed to find associations between BAS/extraversion and indicators of approach motivation obtained from the original EEfRT could be partly due to confounding strategic choices of participants limiting the validity of the original task (Ohmann et al., 2018). Note, however, that additional data of two smaller studies conducted in our laboratory using the modified EEfRT in different contexts hint at a smaller effect size for this association when combined with the current findings (results will be published elsewhere).

The current observation of a main effect of the DA D2 receptor blocker sulpiride on the average number of clicks after controlling for motor performance provides some support for the validity of the modified EEfRT as a measure of DA-based approach motivation. However, it should be noted that we had predicted an increasing rather than a reducing effect of sulpiride on the average number of clicks based on the assumption that the relatively low dose of 200 mg primarily blocks presynaptic autoreceptors, resulting in a reduction of postsynaptic DA activity (Kuroki et al., 1999; Mereu et al., 1983), thus increasing approach motivation. Studies investigating sulpiride’s effects on reward processing and its neurophysiological correlates often use higher dosages of up to 800 mg resulting in predominantly inhibiting effects (Diederen et al., 2017; Ojala et al., 2018; Weber et al., 2016; Eisenegger et al., 2014). However, it might be possible that administering a single dose of sulpiride evokes inhibiting effects even when used in smaller dosages. Furthermore, the effects of sulpiride have been repeatedly observed to depend on complex interactions of various factors, including dosage, time since intake, and incentive context (Chavanon et al., 2013; Mueller et al., 2014). Therefore, the specific setup of our study might have led to the unexpected direction of the observed effect.

A finding that is limiting the validity of the modified EEfRT in the current study is that the effects of sulpiride neither interacted with reward magnitude nor with reward probability, indicating that the inhibiting effect of sulpiride was independent of reward attributes. Previous studies indicated that EEfRT trials with a low probability of reward attainment are most sensitive to pharmacological manipulations of DA (Wardle et al., 2011), manipulation of asymmetric frontal brain activity (Ohmann et al., 2018), trait differences in approach motivation (Geaney et al., 2015), or clinical impairments of approach motivation (e.g., McCarthy et al., 2016; Yang et al., 2014). The current observation that trait BAS and trait extraversion significantly correlated only with the number of clicks within trials with low probability of reward attainment also matches this pattern. One possible explanation for the missing interaction of substance and reward attributes in our study could be a general decrease in effort independent of rewards.

Decision-making tasks like the original EEfRT (Treadway et al., 2009) and comparable tasks differentiate “effort” and “reward” as two (still interdependent) variables that participants can base their decisions on and have shown that increases in DA can lead to effort-specific changes (Le Heron et al., 2018; Filla et al., 2018; Chong et al., 2015) or reward-specific changes (Skvortsova, Degos, Welter, Vidailhet & Pessiglione, 2017) in performance. For example, Le Heron et al. (2018) showed that participants with Parkinson’s disease accept tasks that require higher effort more often, but not tasks with higher rewards when being under DA medication as compared to no DA medication. However, acceptance rate was already very high for high reward conditions (as compared to high-effort conditions) even under no medication – hinting at possible ceiling effects. There are at least two reasons to assume that our modified EEfRT measures changes in approach motivation: first, participants choose to invest a varying amount of clicks depending on the reward attributes introduced in each trial (see Figures 2 and 3), a pattern which is highly comparable to the results of the original EEfRT (e.g., Treadway, et al., 2009; Ohmann et al., 2018). Therefore, we assume that number of clicks invested in each trial is an active decision itself, as each click increases the possible monetary gain. Second, we introduced motoric trials without any rewards before each block of the modified EEfRT and used performance in these to control for motor performance/motoric vigor. Effects of reward magnitude and extraversion/trait BAS were found even with this control.

Unexpectedly, participants within the sulpiride group showed worse performance in the motoric trials. Therefore, it is possible that sulpiride reduced participants’ motoric performance. Nonetheless, the variance introduced via the (reduced) motoric abilities was at least partly controlled by our including performance in the motoric trials in all GEE models (see Section 2.1).

Taken together, the current pattern of results provides initial support for the validity of the modified version of the EEfRT as a measure of approach motivation and also hints at a possible sensitivity of the task to manipulations of DA. However, the limiting factors (global effect of sulpiride and impact of motoric abilities) call for further improvements of the task design. For example, further motoric trials could be introduced before study manipulations, as these could be used to measure participants “baseline” motoric performance. More research with samples at least as large as in the present study is needed to delineate the effects of both trait BAS/extraversion and manipulations of DA levels on approach motivation as measured by the (modified) EEfRT. Until then, the current observation of a reducing effect of 200 mg sulpiride on the number of rewarded clicks in our modified EEfRT only provides limited support for an influence of DA on performance in the task. Nonetheless, we tentatively recommend use of the modified version of the EEfRT to compensate for the limitations of the original task (Ohmann et al., 2018). Note, however, that these limitations may only apply when overall reward in the original EEfRT is based on the total reward amount obtained across all trials (e.g., Hughes et al., 2015) rather than on the reward obtained in two randomly chosen trials (Treadway et al., 2009). The impact of such seemingly subtle task variations is hardly investigated and may often be underestimated.

### 3.2 Conceptual replication of a modulating effect of extraversion on a pharmacological manipulation of DA

In contrast to several previous studies using an identical dose of the same pharmacological agent (e.g., Wacker, 2018; Wacker et al., 2013), we did not observe the expected modulating effect of trait extraversion on the effects of sulpiride. This surprising absence of the expected effect despite encouraging support for the validity of the modified EEfRT and use of a relatively large sample is difficult to interpret. Possibly differences between studies in timing of substance and task administration or incentive context might play a role in this respect (see Section 4.1. Furthermore, we assessed participants’ trait extraversion and might have missed out on substance-induced changes in state extraversion. Such changes in state extraversion might have affected participants’ performance and should be assessed in future studies. Alternatively, the moderating effects of extraversion may be more pronounced for sulpiride’s effects on cognitive control (Wacker, 2018) and working memory (Wacker et al., 2006) than on approach motivation as measured by our modified EEfRT.

Ideally, future studies should either systematically compare various tasks and contexts while controlling for time since administration of various doses of sulpiride or incorporate more direct assessments of sulpiride’s effects in the brain (e.g., using positron emission tomography with appropriate tracers) in order to disentangle effects of time/drug metabolism and task/context variability.

### 3.3 Limitations

Although the present study features a relatively large sample size, controls for a wide range of potential confounding factors, and offers a careful examination of the validity of our modified version of the EEfRT, several limitations should also be mentioned. First, our sample consisted of only male participants aged between 18 and 35 years, most of whom were college students. Therefore, generalizability of our results is necessarily limited. Additionally, we used a between-subject design to counteract possible learning effects between study days. However, a within-subject design could be more sensitive to detect the moderating effect of extraversion. More importantly, small effect sizes seem to be quite common in psychology (Open Science Collaboration, 2015; Gignac & Szodorai, 2016) and we did not find equally strong relationships between BAS/extraversion and behavior in the modified EEfRT in two smaller samples using different contexts (results will be published elsewhere), the present study may still be underpowered and future studies may be well advised to share the load of data collection among several laboratories to achieve sufficient power for more modest effects within a reasonable time frame. Furthermore, although we considered participants’ motoric abilities and accounted for this variance by adding motoric trials to the modified EEfRT, the large impact of motoric abilities may still be considered a possible downside of this task. Future studies should address this limitation.

### 3.4 Conclusions

Taken together, our findings provide initial support for the validity of our modified EEfRT as a measure of approach motivation and suggest that a low dose of sulpiride reduces/impairs approach motivation as measured with the modified EEfRT. As this result was contradictory to our expectations, future studies should consider investigating various possible moderating factors (study design, incentive context, etc.) and limiting factors (global effect of sulpiride and impact of motoric abilities). Nonetheless, the associations between task performance and both extraversion and trait BAS provide tentative support for the hypothesized link between these traits and approach motivation. The unexpected lack of a moderating effect of extraversion on the effects of sulpiride observed in several previous studies (e.g., Wacker, 2018; Wacker et al., 2013) encourages further work aimed at identifying the relevant boundary conditions and further elucidating the hypothesized dopaminergic mechanisms underlying stable individual differences in approach motivation. Future studies should also further improve assessment of participants’ motoric abilities to better delineate approach motivation and motoric abilities within the modified EEfRT, which have shown to strongly impact performance on this task.
